# Circulating SCUBE1 levels in women with polycystic ovary syndrome

**DOI:** 10.4274/tjod.25826

**Published:** 2018-09-03

**Authors:** Onur Erol, Hamit Yaşar Ellidağ, Mustafa Kemal Özel, Aysel Uysal Derbent, Esin Eren, Necat Yılmaz

**Affiliations:** 1 University of Health Sciences, Antalya Training and Research Hospital, Clinic of Obstetrics and Gynecology, Antalya, Turkey; 2 University of Health Sciences, Antalya Training and Research Hospital, Clinic of Biochemistry, Antalya, Turkey

**Keywords:** Platelet activation, Polycystic ovary syndrome, SCUBE1

## Abstract

**Objective:**

Polycystic ovary syndrome (PCOS) is thought to represent an early manifestation of metabolic syndrome, which is associated with cardiovascular disease. Signal peptide-CUB (complement C1r/C1s, Uegf, and Bmp1)-epidermal growth factor domain-containing protein 1 (SCUBE1) is a platelet activation marker that plays important roles in vascular biology and has been closely linked to cardiovascular events. In the present study, we investigated SCUBE1 levels in lean glucose-tolerant women with PCOS and assessed the possible association between SCUBE1 levels and hormonal and metabolic features of women with PCOS.

**Materials and Methods:**

The study population consisted of 90 lean [body mass index (BMI) <25 kg/m^2^] women who were diagnosed as having PCOS using the Rotterdam criteria and 100 age- and BMI-matched healthy controls with no clinical or biochemical feature of hyperandrogenism. Glucose tolerance was evaluated in all subjects before recruitment using the 2 h 75 g oral glucose tolerance test, and only those exhibiting normal glucose tolerance were enrolled. Hormonal and metabolic parameters, and serum SCUBE1 levels were evaluated.

**Results:**

Circulating SCUBE1 levels were significantly higher in women with PCOS than in controls (5.9±3.9 vs. 4.2±1.4 ng/mL, p=0.022). No association between SCUBE1 level and clinical or biochemical parameters was found in the control or PCOS group.

**Conclusion:**

SCUBE1 levels are elevated in women with PCOS compared with those in healthy controls; thus, this protein may be an early biomarker of cardiovascular disease later in life.

**PRECIS:** Circulating SCUBE1 levels are elevated in women with Polycystic ovary syndrome compared with those in healthy controls; thus, this protein may be an early biomarker of cardiovascular disease later in life.

## Introduction

Polycystic ovary syndrome (PCOS) is one of the most common endocrine disorders in women of reproductive age, and is a heterogeneous clinical condition characterized by hyperandrogenism and signs of chronic oligo-/anovulation. The prevalence of PCOS ranges from 6% to 10%, depending on the criteria used, ethnicity, and geographic location^(1)^. Although originally considered to be a gynaecologic disorder, PCOS is associated with reproductive and metabolic disturbances, including ovulatory dysfunction, hyperandrogenism, dyslipidaemia, increased insulin resistance, and impaired glucose intolerance. Furthermore, PCOS is thought to represent an early manifestation of metabolic syndrome, which is associated with cardiovascular disease^(2)^. Several cardiovascular risk factors, such as dyslipidaemia, impaired fibrinolysis, chronic low-grade inflammation, endothelial dysfunction, and subclinical and clinical atherosclerosis, are more prevalent in women with PCOS^(3)^. In addition, these risks factors are strongly linked to insulin resistance and are compounded by the common occurrence of obesity. Data regarding the long-term cardiovascular consequences of PCOS are conflicting^(4)^.

Signal peptide-CUB (complement C1r/C1s, Uegf, bone morphogenetic protein-1)-epidermal growth factor-like protein (SCUBE) is an evolutionarily conserved gene family composed of three different isoforms (SCUBE1-3)^(5)^. SCUBE family members are secretory membrane proteins that play important roles during mouse embryogenesis by regulating extracellular signal transport, molecular adhesion, and migration^(6,7)^. Although SCUBE1 was originally isolated from vascular endothelial cells, it is stored predominantly in alpha granules of inactive platelets in humans^(8,9)^. Following platelet activation, SCUBE1 expression is up-regulated and SCUBE1 is translocated to the cell surface, cleaved, and released into circulation in small soluble particles. These circulating fragments enhance platelet-platelet adhesion and agglutination under thrombotic conditions. SCUBE1 accumulates in platelet-rich thrombus and atherosclerotic vascular lesions^(9)^. An experimental study showed that genetic loss or functional neutralisation of soluble SCUBE1 prevents thrombosis^(10)^. In addition, a single nucleotide polymorphism of the *SCUBE1* gene is associated with enhanced risk of venous thromboembolism^(11)^. Taken together, these data suggest that SCUBE1 is involved in modulating vascular biology. In this study, we aimed to investigate SCUBE1 levels in lean glucose-tolerant women with PCOS, and to assess the possible associations between SCUBE1 levels and the hormonal and metabolic features of this syndrome.

## Materials and Methods

This prospective case-control study was conducted at the Antalya Training and Research Hospital, Antalya, Turkey, between June 2015 and March 2016. The Ethics Committee of the institution approved this study, and all subjects provided written informed consent. A total of 190 lean [body mass index [BMI] <25 kg/m^2^] patients, aged 20-35 years,withnormal glucose tolerance (NGT), were recruited from the outpatient gynaecology clinic of our institution. The study group consisted of 90 women diagnosed with PCOS using the revised 2003 Rotterdam consensus criteria as the presence of two of the following three features: 1) oligomenorrhea (inter-menstrual interval >35 days) or amenorrhea (absence of menstruation for 3 consecutive months), 2) clinical and/or biochemical signs of hyperandrogenism, and 3) polycystic ovaries as revealed by typical imaging features on ultrasonographic examination (12 or more follicles 2-9 mm in diameter in each ovary and/or ovarian volume >10 cm^3^)^(12)^. One hundred healthy women with no clinical or biochemical features of hyperandrogenism were recruited as the control group. All of the control subjects were ovulatory as evidenced by regular menstruation (lasting 21 to 35 days) and luteal-phase serum progesterone levels >5 ng/mL. The PCOS and control groups were matched in terms of both age and BMI. Glucose tolerance was evaluated before study recruitment using the criteria of the American Diabetes Association^(13)^. Thus, the 2 h 75 g oral glucose tolerance test (OGTT) was administered to all subjects, and only those with NGT were enrolled. NGT was defined as a fasting glucose level <100 mg/dL or a 2 h glucose level <140 mg/dL. Exclusion criteria were impaired glucose regulation, diabetes mellitus (DM), hyperprolactinemia, thyroid dysfunction, Cushing’s syndrome, congenital adrenal hyperplasia, acromegaly, hypothalamic disorder, hypertension, systemic inflammatory disease, any vascular disorder, coagulation abnormalities, history of alcohol consumption or smoking, and family history of DM and/or PCOS. No participant had taken any medication known to affect hormone, lipid, or carbohydrate metabolism (e.g. insulin-sensitising drugs, oral contraceptives, anti-androgens, corticosteroids, statins, or aspirin) within the previous 3 months. Late-onset congenital adrenal hyperplasia was excluded by measuring a normal   17-hydroxyprogesterone level (<1.2 ng/mL during the early follicular phase) in a baseline morning blood sample.

### Anthropomorphic and clinical measurements

As part of the physical examination, weight and height of each patient were recorded, and BMI was calculated using the formula: weight (kg)/height (m^2^). Waist and hip circumferences were measured in a standing position with the feet fairly close together. Waist circumference was measured midway between the lower rib margin and the iliac crest, and hip circumference was measured over the maximum circumference of the buttocks, to calculate the waist/hip ratio (WHR). Systolic and diastolic blood pressure of each patient was measured after a 10 min rest and recorded. The modified Ferriman-Gallwey figure was self-scored after all participants were given an explanation and demonstration in full detail^(14)^.Hyperandrogenism was defined as clinical hirsutism (modified Ferriman-Gallwey score ≥8), acne, alopecia, and/or an elevated androgen level (total testosterone >0.75 ng/mL (manufacturer’s reference range: 0.1-0.75 ng/mL) and/or dehydroepiandrosterone sulphate (DHEAS) >430 µg/dL (manufacturer’s reference range: 35-430 µg/dL). Ovarian morphology was evaluated by transvaginal ultrasonography or transabdominal ultrasonography (DC-7, Mindray Medical International Ltd., Shenzhen, China) with a distended bladder in virginal women on the same day that blood samples were obtained.

### Biochemical Analysis

Laboratory tests were performed during the early follicular phase (days 3-7 of the menstrual cycle) after a spontaneous bleeding episode, or independent of the cycle phase if amenorrhea was evident. Baseline blood samples were obtained from large forearm antecubital veins after a 12 h overnight fast, and all subjects underwent the standard 2 h 75 g OGTT. The blood samples were placed in plain tubes, stored at room temperature for at least 30 min to allow clotting, and centrifuged at 2500 rpm for 15 min at 4 °C to separate the serum. Concentrations of serum glucose, insulin, and other hormone and lipid parameters were assayed immediately. Additional serum was isolated from fasting blood samples and stored at -80 °C for later analysis of SCUBE1 level. Serum follicle-stimulating hormone (FSH), luteinizing hormone (LH), total testosterone, and sex hormone-binding globulin (SHBG) levels were determined using a two-site immunoenzymatic method, and DHEAS levels were measured using a competitive binding immunoenzymatic method employing a commercially available kit (Beckman Coulter Diagnostics, Fullerton, CA, USA) and an autoanalyer (Access DxI800; Beckman Coulter). Serum 17-hydroxyprogesterone levels were determined using a commercially available kit (DiaMetra, Segrate, Italy) and an autoanalyzer (Etimax 3000; DiaSorin, Stillwater, MN, USA). Glucose levels were measured using the hexokinase technique and a commercially available kit (Beckham AU5800; Beckham Coulter). Insulin levels were determined using a chemiluminescent assay (AccessDxI800; Beckman Coulter). Serum triglyceride, total cholesterol, high-density lipoprotein, and low-density lipoprotein cholesterol levels were determined using an autoanalyzer (Beckman AU5800; Beckman Coulter). The intra- and inter-assay coefficients of variation (CVs) for all assays were 5% and 10%, respectively.

The free androgen index (FAI) was calculated as total serum testosterone level (nmol/L) ´100/SHBG (nmol/L). We estimated insulin resistance using the homeostatic model assessment-insulin resistance (HOMA-IR) index, defined as fasting plasma insulin value (µU/mL) ´ fasting plasma glucose value (mg/dL)/405^(15)^. Insulin sensitivity was calculated using the quantitative insulin-sensitivity check index (QUICKI), according to the following formula: 1/[log (fasting insulin level (µU/mL) + log (fasting glucose level (mg/dL)]^(16)^.

Serum SCUBE1 levels were measured using a commercially available enzyme-linked immunosorbent assay (cat no. E-EL-H5405; Elabscience Biotechnology, Wuhan, China), according to the manufacturer’s instructions. Assay sensitivity was 0.38 ng/mL, and the inter- and intra-assay CVs were <10% and 8%, respectively.

### Statistical Analysis

The normality of data distribution was assessed using the Kolmogorov-Smirnov test. Continuous variables are presented as mean ± standard deviation if normally distributed or as median (range) if not normally distributed. Between-group differences were detected using Student’s t-test for parametric data and the Mann-Whitney U test for nonparametric data. Correlations between SCUBE1 levels and other parameters were calculated using Pearson’s correlation analysis (normally distributed data) or Spearman’s rank test (data not normally distributed). Two-sided p values <0.05 were considered to be significant. The statistical analysis was performed using the SPSS ver. 18.0 software (SPSS Inc., Chicago, IL, USA).

## Results

The clinical characteristics and biochemical data of the control subjects and patients with PCOS are presented in Table 1. These parameters were similar between the two groups because the participants were matched in terms of age and BMI (p>0.05). As expected, the hirsutism score was significantly higher in patients with PCOS than in the control group (p<0.001). Obstetric history and WHR did not differ between the groups (p>0.05). Serum levels of LH and total testosterone, as well as the FAI were significantly higher, but serum SHBG level was significantly lower in women with PCOS than in the control group (p<0.05 for all). On the other hand, no differences in FSH, 17-hydroxyprogesterone, DHEAS, insulin, fasting or 2 h post-load glucose concentration, HOMA-IR or QUICKI values or lipid parameters were detected between the two groups. Serum SCUBE1 levels were significantly higher in patients with PCOS than in the controls (5.9±3.9 vs. 4.2±1.4 ng/mL, p=0.022). Serum SCUBE1 levels in patients with PCOS stratified according to hyperandrogenism were not statistically different from one another (5.8±2.8 ng/mL in normoandrogenic PCOS vs. 5.9±3.1 ng/mL in hyperandrogenic PCOS, p=0.91). No significant correlation was found between SCUBE1 concentrations and any clinical or biochemical parameters in either group (Table 2).

## Discussion

Our results show that serum levels of SCUBE1, a platelet activation marker, were significantly higher in young, lean glucose-tolerant women with PCOS than in age- and BMI-matched healthy controls. Moreover, no significant correlations were detected between any hormonal or metabolic PCOS parameters and SCUBE1 concentrations. These results suggest that PCOS, in the absence of obesity and glucose intolerance, results in increased platelet activation, even in young women. To the best of our knowledge, this is the first study to evaluate SCUBE1 levels in women with PCOS. Although platelets are involved in fundamental processes of vascular biology, excess platelet activation may lead to platelet-mediated thrombosis and associated clinical ischemic events. Several studies have investigated SCUBE1 as a marker of platelet activation in such patients. Dai et al^(17)^. reported that SCUBE1 was elevated in patients with acute coronary syndrome and acute ischaemic stroke compared with patients with chronic coronary disease and healthy controls. These findings were corroborated by Sonmez et al.,^(18)^ who reported that the analysis of circulating SCUBE1 levels provided useful diagnostic information to distinguish patients with acute coronary syndrome from those with non-coronary chest pain. Given the relationship between platelet hyperactivity and cardiovascular events, extensive interest has developed regarding platelet function in women with PCOS. Dereli et al.^(19) ^demonstrated higher platelet aggregation induced by adenosine diphosphate (ADP), collagen, and epinephrine in a cohort of lean women with PCOS compared with those in age-and BMI-matched controls. Of interest, platelet aggregation was negatively correlated with insulin sensitivity. Rajendran et al.^(20)^ found more ADP-induced platelet aggregation and less platelet responsiveness to the inhibitory effects of nitric oxide in lean and obese women with PCOS than in age-matched controls. These authors suggested that hyperandrogenism was responsible for impaired platelet function in women with PCOS because no difference in platelet aggregation was demonstrated between the lean and obese PCOS groups. In contrast, Kahal et al.^(21)^ found no difference in baseline platelet function, ADP-induced platelet aggregation, or platelet responsiveness to the inhibitory effects of prostacyclin between obese women with PCOS and BMI-matched controls. These equivocal data can be explained by the use of different PCOS diagnostic criteria and techniques to assess platelet function. In addition to the *in vitro* studies mentioned above, several research groups have investigated *in vivo* platelet activation biomarkers, such as P-selectin and platelet-derived microparticles (PMPs), in patients with PCOS. P-selectin is an adhesion molecule secreted extracellularly from platelet alpha granules that is involved in platelet aggregation. Yildiz et al.^(22)^ demonstrated that soluble P-selectin levels were significantly higher in young, normal glucose-tolerant women with PCOS than in age-and BMI-matched healthy controls. On the other hand, no significant correlation was observed between soluble P-selectin levels and any anthropometric or biochemical parameter in the PCOS group. PMPs are small vesicles released from the surface of activated or apoptotic platelets as a result of membrane remodelling. PMPs are highly procoagulant due to expression of phospholipids and tissue factors on their outer membranes, which are the main initiators of the coagulation cascade. Koiou et al.^(23,24)^ reported higher circulating PMP levels in lean and overweight/obese women with PCOS compared with those in BMI-matched controls. PMPs were correlated with serum testosterone levels^ (23) ^and the mean number of ovarian follicles^(24)^.

In the present study, serum total testosterone levels and FAI were significantly higher in women with PCOS than in controls. Therefore, we speculate that higher SCUBE1 levels in women with PCOS may be attributable to hyperandrogenaemia. However, we failed to show any correlation between SCUBE1 and total testosterone level, FAI, or any other clinical or biochemical parameter in either the control or PCOS groups. The reason for these results is linked to the fact that we included only lean and normal glucose-tolerant subjects to avoid any confounding effects of obesity and/or impaired glucose tolerance on platelet function^(25,26)^. Low-grade systemic inflammatory activation in patients with PCOS may contribute, at least in part, to increased SCUBE1 level^(27)^. Indeed, SCUBE1 levels increase in response to stimulation by proinflammatory cytokines, such as interleukin-1b and tumour necrosis factor-α^(8)^. Taken together, these findings suggest that increased SCUBE1 level is an independent contributor to an increased risk of cardiovascular events in women with PCOS, regardless of other traditional risk factors, such as obesity, insulin resistance, and hyperandrogenism.

### Study Limitations

The present study has several limitations. Our study design was cross-sectional in nature, and long-term consequences of increased SCUBE1 level in women with PCOS were not evaluated. Another limitation is that although SCUBE1 is derived mainly from platelets; we did not investigate expression of this marker from those cells. Finally, our study subjects were mostly young women; therefore, our results may not be generalizable to older patients with PCOS.

## Conclusion

Circulating SCUBE1 levels are elevated in young, lean, glucose-tolerant women with PCOS compared with those in healthy controls; thus, this protein may be an early biomarker of cardiovascular disease later in life. Additional studies are required to clarify the potential impact of SCUBE1 in the pathogenesis of PCOS and to investigate its association on the cardiovascular risk of these patients.

## Figures and Tables

**Table 1:**
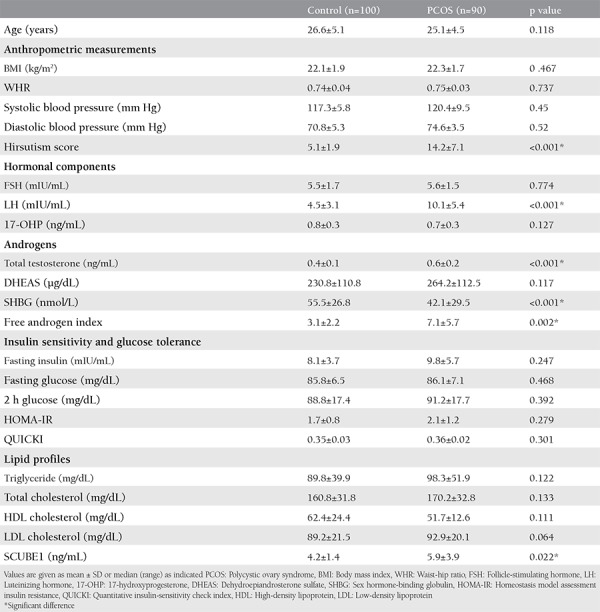
Clinical and laboratory features of the control and Polycystic ovary syndrome groups

**Table 2:**
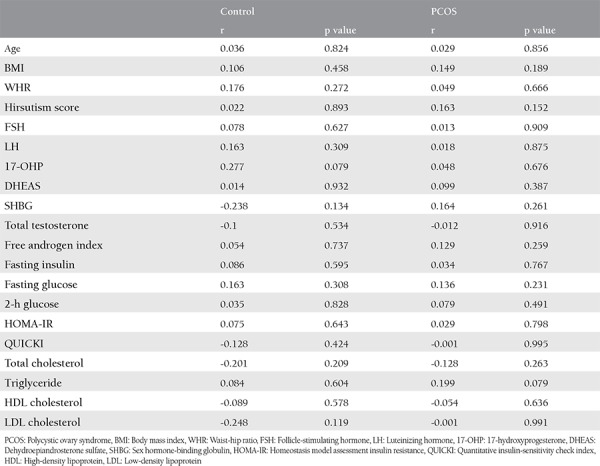
Correlations of SCUBE1 levels with clinical and biochemical parameters in the control and Polycystic ovary syndrome groups
